# Long non-coding RNA HULC promotes tumor angiogenesis in liver cancer by up-regulating sphingosine kinase 1 (SPHK1)

**DOI:** 10.18632/oncotarget.6280

**Published:** 2015-11-02

**Authors:** Zhanping Lu, Zelin Xiao, Fabao Liu, Ming Cui, Weiping Li, Zhe Yang, Jiong Li, Lihong Ye, Xiaodong Zhang

**Affiliations:** ^1^ The State Key Laboratory of Medicinal Chemical Biology, Department of Cancer Research, College of Life Sciences, Nankai University, Tianjin, P.R. China; ^2^ The State Key Laboratory of Medicinal Chemical Biology, Department of Biochemistry, College of Life Sciences, Nankai University, Tianjin, P.R. China; ^3^ Department of Medical Science Laboratory, Fenyang College, Shanxi Medical University, Fenyang, Shanxi Provence, P.R. China

**Keywords:** HULC, HCC, SPHK1, angiogenesis, E2F1, miR-107

## Abstract

Highly up-regulated in liver cancer (HULC) is a long non-coding RNA (lncRNA). We found that HULC up-regulated sphingosine kinase 1 (SPHK1), which is involved in tumor angiogenesis. Levels of HULC were positively correlated with levels of SPHK1 and its product, sphingosine-1-phosphate (S1P), in patients HCC samples. HULC increased SPHK1 in hepatoma cells. Chicken chorioallantoic membrane (CAM) assays revealed that si-SPHK1 remarkably blocked the HULC-enhanced angiogenesis. Mechanistically, HULC activated the promoter of SPHK1 in hepatoma cells through the transcription factor E2F1. Chromatin immunoprecipitation (ChIP) and electrophoretic mobility shift assay (EMSA) further showed that E2F1 was capable of binding to the E2F1 element in the SPHK1 promoter. HULC increased the expression of E2F1 in hepatoma cells and levels of HULC were positively correlated with those of E2F1 in HCC tissues. Intriguingly, HULC sequestered miR-107, which targeted E2F1 mRNA 3′UTR, by complementary base pairing. Functionally, si-SPHK1 remarkably abolished the HULC-enhanced tumor angiogenesis *in vitro* and *in vivo*. Taken together, we conclude that HULC promotes tumor angiogenesis in liver cancer through miR-107/E2F1/SPHK1 signaling. Our finding provides new insights into the mechanism of tumor angiogenesis.

## INTRODUCTION

Long non-coding RNAs (lncRNAs) are transcripts with no protein coding function, which are longer than 200 nucleotides [1]. The dysregulation of lncRNAs gradually becomes a primary feature of human cancers, such as melanoma [2], gastric cancer[3], bladder cancer [4] and hepatocellular carcinoma (HCC) [5]. Highly up-regulated in liver cancer (HULC), as a lncRNA, is correlated with the development of HCC [6-8]. HULC can be up-regulated by CREB through sequestering miRNA-372 [9]. Our group reported that the HBx-elevated HULC promoted hepatoma cell proliferation via decreased p18 [10] and increased abnormal lipid metabolism in hepatoma cells through a miRNA-9 mediated RXRA signaling pathway [11].

The rapid growth of cancer requires a blood supply [12]. To obtain the blood supply, tumor cells can tilt the balance toward activating angiogenic factors, such as vascular endothelial growth factor (VEGF) and fibroblast growth factor (FGF) families, to drive angiogenesis [13, 14]. Meanwhile, angiogenesis can help tumor cells to spread with the blood stream to distant organs [15, 16]. Therefore, the angiogenesis plays a crucial role in the tumor development and metastasis. However, the role of lncRNA HULC in tumor angiogenesis remains unclear.

Sphingosine-1-phosphate (S1P) is able to promote cell survival [17], proliferation [18], differentiation and angiogenesis [19]. Sphingosine kinase 1 (SPHK1) is a conservative enzyme which generates S1P, and takes part in the regulation of the sphingolipid biostat [20]. It has been reported that SPHK1-produced S1P promotes breast cancer progression by stimulating angiogenesis [19]. Sphingosine kinase/S1P/S1P receptor axis has been reported to be involved in liver fibrosis-associated angiogenesis [21]. However, whether HULC is involved in the activation of SPHK1 in HCC is ill-defined.

In the present study, we seek to investigate the role of lncRNA HULC in angiogenesis of liver cancer. Interestingly, our data show that HULC is capable of promoting tumor angiogenesis through miR-107/E2F1/SPHK1 signaling in hepatoma cells. Our finding provides new insights into the mechanism of liver cancer angiogenesis.

## RESULTS

### HULC contributes to the angiogenesis in liver cancer by up-regulating SPHK1

**Figure 1:**
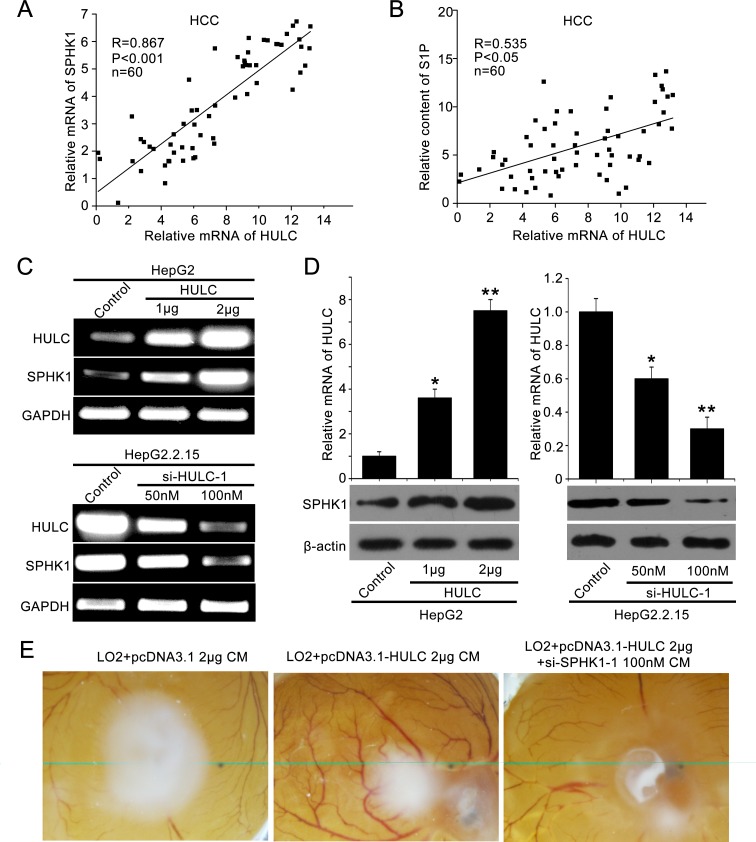
HULC contributes to angiogenesis in liver cancer by up-regulating SPHK1 **A.** The correlation between SPHK1 mRNA levels and HULC mRNA levels was detected by qRT-PCR in 60 HCC tissues (*P* < 0.001, *r* = 0.867, Pearson's correlation). **B.** The correlation between S1P and HULC was detected by ELISA and qRT-PCR in above HCC tissues (*P* < 0.05, *r* = 0.535, Pearson's correlation). **C.** The mRNA expression of SPHK1 was examined by RT-PCR after transfected with the pcDNA3.1-HULC plasmid (or si-HULC-1) in HepG2 (or HepG2.2.15) cells. The transfection efficiency of HULC (or si-HULC-1) was detected by RT-PCR. **D.** The expression of SPHK1 was examined by Western blot analysis after transfection of pcDNA3.1-HULC plasmid (or si-HULC-1) in HepG2 (or HepG2.2.15) cells. The transfection efficiency of HULC (or si-HULC-1) was detected by qRT-PCR. **E.** Representative images of chicken chorioallantoic membrane blood vessels stimulated with conditioned medium (see Materials and Methods).

To investigate the role of HULC in tumor angiogenesis, we screened the expression of several tumor angiogenesis-associated factors, such as VEGF, SPHK2, transforming growth factor-beta receptor 2 (TGF-βR2), monocyte chemoattractant protein 1 (MCP1) and SPHK1 [22, 23], in HULC over-expressed HepG2 cells. Interestingly, we observed that HULC remarkably increased SPHK1 at the levels of mRNA in a dose-dependent manner (supporting Figure 1A). Then, we detected the relationship of HULC and SPHK1 in clinical HCC tissues. Our data showed that the expression levels of HULC were positively correlated with those of SPHK1 (or S1P) by qRT-PCR (or ELISA) in 60 clinical HCC samples (*P* < 0. 001, *r* = 0.867; *P* < 0. 005, *r* = 0.535, Pearson's correlation) (Figure 1A, 1B). It has been reported that the mRNA levels of SPHK1 are increased in liver cancer tissues [24]. In this study, we further assessed the protein levels of SPHK1 in 143 HCC samples using tissue arrays. Immunohistochemical (IHC) staining revealed that the strong positive staining of SPHK1 was 98 out of 143 (68.53%) in clinical HCC tissues (supporting Table 2 and supporting Figure 1B). Moreover, we showed that the expression levels of SPHK1 mRNA and protein were increased by HULC in HepG2 cells in a dose-dependent manner (Figure 1C and 1D). The same event was observed in other hepatoma cells, such as Huh7 and H7402 (supporting Figure 1D and 1E). To block the function of HULC, we synthesized two siRNAs which might target HULC. The interfering efficiency of the two siRNAs was examined by qRT-PCR in HepG2 cells (supporting Figure 1C). As expected, si-HULC-1 and si-HULC-2 could efficiently decrease the expression of SPHK1 at both mRNA and protein levels (Figure 1C and 1D and supporting Figure 1F and 1G). In addition, we measured the levels of S1P in the medium of HepG2 (or HepG2.2.15) cells transfected with pcDNA3.1-HULC (or si-HULC-1) by ELISA. We found that overexpression of HULC (or depletion of HULC) could enhance (or decrease) the secretion of S1P from hepatoma cells into the medium by SPHK1 (supporting Figure 1H). We further evaluated the pro-angiogenesis of HULC using CAM assays. Our data exhibited that the conditional medium from HULC-overexpressed HepG2 cells remarkably promoted the angiogenesis of chicken chorioallantoic membrane, which could be efficiently blocked by SPHK1 siRNA (Figure 1E). Additionally, the interfering efficiency of si-SPHK1-1 and si-SPHK1-2 was detected by Western blot analysis in HepG2 cells. Accordingly, si-SPHK1-1 was selected for further study (supporting Figure 1I). Thus, we conclude that lncRNA HULC might be related to the angiogenesis in liver cancer by the up-regulation of SPHK1.

### HULC activates SPHK1 promoter through transcription factor E2F1

**Figure 2:**
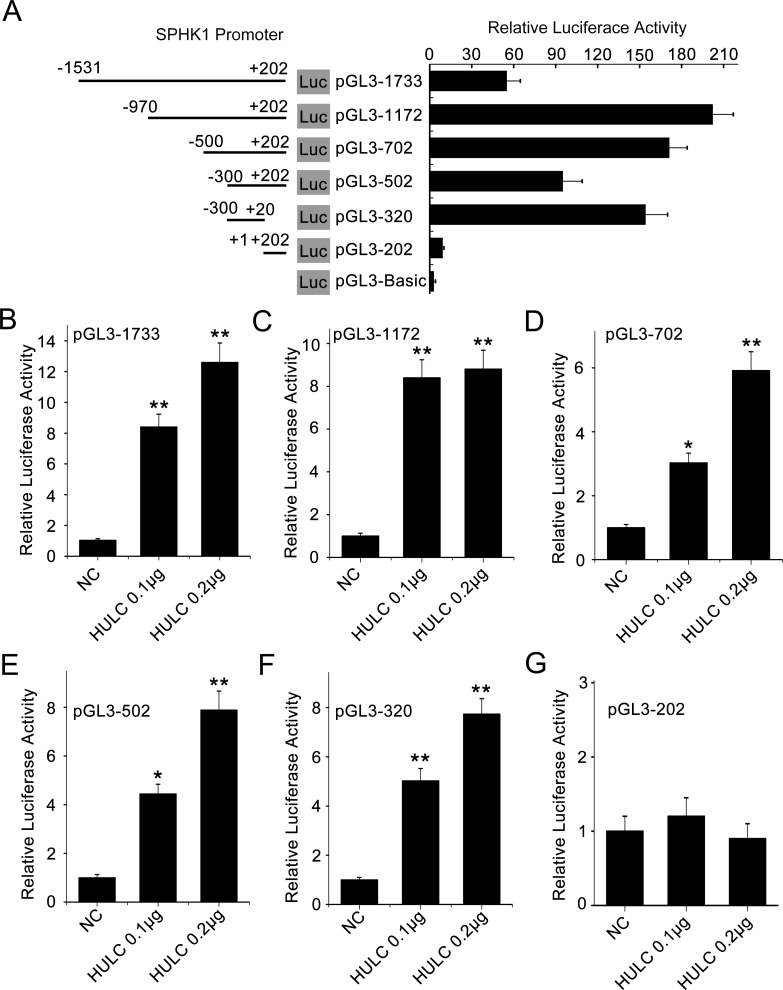
HULC is able to activate the promoter of SPHK1 **A.** HepG2 cells were transiently transfected with pGL3-Basic (0.2 μg/well) or reporter constructs containing various lengths of the 5′-flanking region of the SPHK1 gene, as indicated (pGL3-1733, pGL3-1172, pGL3-702, pGL3-502, pGL3-320 and pGL3-202, 0.2 μg/well, respectively. Results were obtained as relative luciferase activities against the activity of pGL3-Basic. **B.**-**G.** HepG2 cell lines were co-transfected with reporter constructs (0.2 μg/well) and HULC expression plasmid (pcDNA3.1-HULC), respectively. Promoter activities of SPHK1 were measured by luciferase reporter gene assays. Data are shown as mean ± SD of three independent experiments. *P < 0.05; **P < 0.01; Student's *t* test.

To unravel the underlying mechanism by which HULC increases SPHK1, we focused on the investigation in transcriptional regulation of SPHK1. Various fragments of the SPHK1 5′ flanking region, including −1531/+202 (pGL3-1733), −970/+202 (pGL3-1172), −1531/−951 (pGL3-581), −500/+202 (pGL3-702), −300/+202 (pGL3-502), −300/+20 (pGL3-320) and +1/+202 (pGL3-202), were cloned and transiently transfected into HepG2 (or 293T) cells, respectively. Luciferase reporter gene assays indicated that the fragment pGL3-320 exhibited the highest luciferase activities among the various regions (Figure 2A and supporting Figure 2A), indicating that the region of −300/+20 is the core region of SPHK1 promoter. Then we validated that HULC could increase the luciferase activities of pGL3-1733, pGL3-1172, pGL3-702, pGL3-502 and pGL3-320 in HepG2 or 293T cell lines in a dose-dependent fashion, respectively (Figure 2B-2F and supporting Figure 2B and 2D-2G). However, HULC failed to work for activating pGL3-202 (Figure 2G) and pGL3-581 (supporting Figure 2C). Thus, we conclude that HULC is able to activate the core region of SPHK1 promoter.

**Figure 3:**
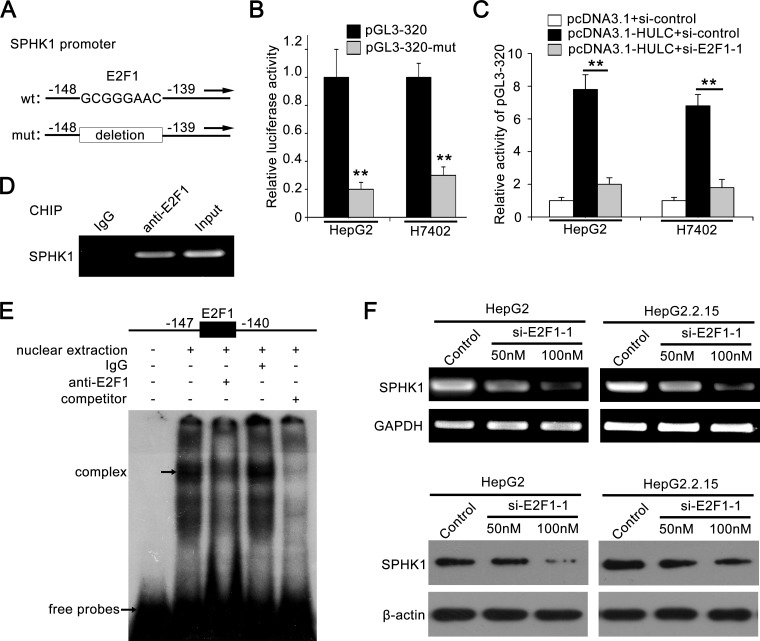
Transcription factor E2F1 activates SPHK1 promoter by binding to the promoter region **A.** A model demonstrates the predicted conserved E2F1 binding site at nucleotides −147/−140 of the SPHK1 promoter. The generated mutant site at the SPHK1 promoter region is indicated. The mutant type was inserted into pGL3-Basic vector. **B.** The luciferase activities of pGL3-320 (or pGL3-320-mut) were detected in HepG2 (or H7402) cells. **C.** Relative activity of pGL3-320 was measured in HepG2 (or H7402) cells transfected with HULC or cotransfected with HULC and si-E2F1-1. **D.** ChIP assays were performed in HepG2 cells to confirm the interaction of E2F1 and SPHK1 promoter region. **E.** Interaction between E2F1 and SPHK1 promoter region was examined by EMSA. **F.** The expression levels of SPHK1 were detected by RT-PCR and Western blot analysis in HepG2 and HepG2.2.15 cells transfected with si-E2F1-1, respectively. Data are shown as mean ± SD of three independent experiments. *P < 0.05, **P < 0.01, Student's *t* test.

Using the bioinformatic tool, we observed an E2F1 binding site in the region −147 to −140 by analyzing the core region of SPHK1 promoter (http://alggen.lsi.upc.edu). It has been reported that E2F1 plays an important role in tumor angiogenesis [25, 26]. Thus, we focused on the investigation of E2F1 function in HULC-promoted angiogenesis. In addition, we constructed the mutant of SPHK1 promoter, termed pGL3-320-mut, with deletion of eight nucleotides in E2F1 binding site (Figure 3A). As expected, we observed that the relative luciferase activities of pGL3-320-mut were obviously decreased compared to those of pGL3-320 (Figure 3B). Moreover, E2F1 siRNA significantly abolished the activation of SPHK1 promoter increased by HULC (Figure 3C), suggesting that E2F1 is involved in the activation of SPHK1 promoter increased by HULC. Interference efficiency of si-E2F1-1 and si-E2F1-2 was detected in HepG2 cells by Western blot analysis (supporting Figure 3A). ChIP assay showed that E2F1 was able to interact with the SPHK1 promoter in HepG2 cells and HCC tissues (Figure 3D and supporting Figure 3B). As shown in Figure 3E, the interaction between nuclear proteins and SPHK1 promoter sequences was observed by electrophoretic mobility shift assays (EMSA). The specific binding of nuclear protein to the ^32^P-labeled SPHK1 promoter was decreased when cold competitor or anti-E2F1 antibody (Ab) was added into the nuclear extracts. In addition, we further confirmed that E2F1 siRNA could decrease the expression of SPHK1 at the levels of mRNA and protein in HepG2 or HepG2.2.15 cells in a dose-dependent manner (Figure 3F and supporting Figure 3C). In addition, we examined the expression of E2F1 in above 143 HCC samples using tissue arrays. Immunohistochemical (IHC) staining revealed that the strong positive staining of E2F1 was 121 out of 143 (84.62%) (supporting Figure 3D and supporting Table 2). Taken together, we conclude that the transcription factor E2F1 is responsible for the activation of SPHK1 promoter elevated by HULC.

### HULC is able to up-regulate E2F1 by sequestering miR-107

**Figure 4:**
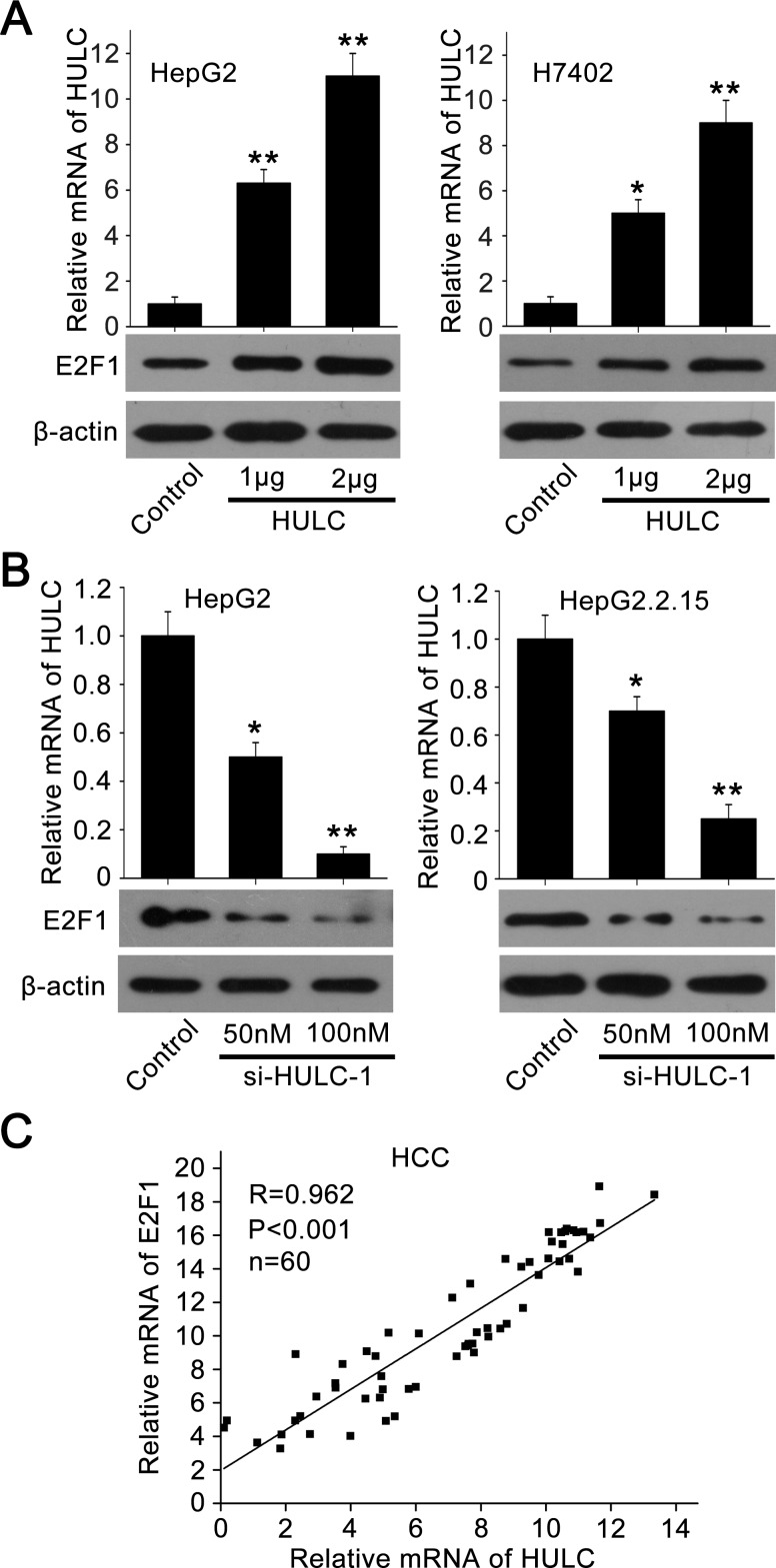
HULC is able to increase the expression of E2F1 **A.**, **B.** The expression of E2F1 was examined by Western blot analysis in HepG2 and H7402 (or HepG2.2.15) cells transfected with pcDNA3.1-HULC (or si-HULC-1). The transfection efficiency of HULC (or si-HULC-1) was detected by qRT-PCR. **C.** The correlation between E2F1 mRNA levels and HULC mRNA levels was detected by qRT-PCR in 60 HCC tissues (*P* < 0.001, r = 0.962, Pearson's correlation). *P < 0.05; **P < 0.01; Student's *t* test.

To investigate the relationships between HULC and E2F1, we evaluated the expression levels of E2F1 in hepatoma cells. Interestingly, Figure 4A showed that HULC was able to increase E2F1 in HepG2 and H7402 cells in a dose-dependent manner. But si-HULC-1 displayed the reverse event (Figure 4B). Moreover, we observed that the expression levels of HULC were positively related to those of E2F1 in above 60 HCC tissues (*P* < 0. 001, *r* = 0.962, Pearson's correlation) (Figure 4C).

**Figure 5:**
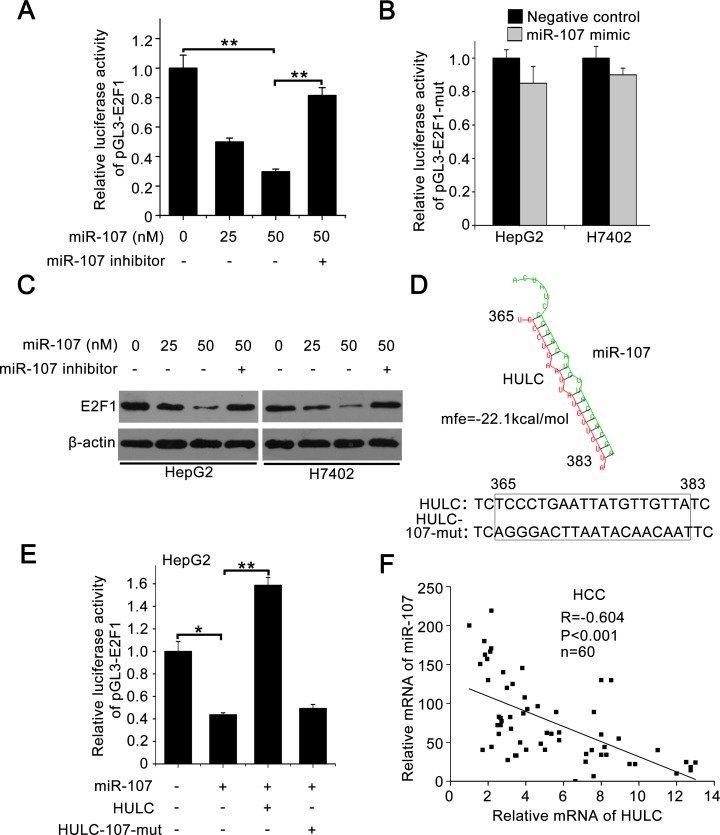
HULC increases E2F1 by sequestering miR-107 **A.** Relative luciferase activity of pGL3-E2F1 was measured by luciferase reporter gene assays in HepG2 cells transfected with miR-107 or co-transfected with miR-107 inhibitor. **B.** Relative luciferase activities of pGL3-E2F1-mut were detected in HepG2 and H7402 cells treated with miR-107. **C.** E2F1 was measured by Western blot analysis in HepG2 and H7402 cells transfected with miR-107 or co-transfected with miR-107 and miR-107 inhibitor. **D.** A model shows the predicted interaction between HULC and miR-107 through complementary base-pairs. The generated mutant site at the HULC (365-383) is indicated. **E.** Relative luciferase activities of pGL3-E2F1 were measured by luciferase reporter gene assays in HepG2 cells transfected with miR-107 or co-transfected with HULC (or HULC-107-mut). **F.** The correlation between HULC mRNA levels and miR-107 levels was detected by qRT-PCR in 60 HCC tissues (*P* < 0.001, r = −0.604, Pearson's correlation). Data are shown as mean ± SD of three independent experiments. *P < 0.05, **P < 0.01, Student's *t* test.

To better understand the mechanism, we analyzed the 3′ untranslated region (3′UTR) of E2F1 mRNA using the website software (http://diana.imis.athena-innovation.gr/DianaTools/index.php). We found that three miRNAs might bind the 3′UTR of E2F1 mRNA, including miR-107, miR-135 and miR-208b. However, miR-135 had little effect on the expression of E2F1 (data not shown). MiR-208b could not be expressed in the liver cancer [27]. Therefore, we selected miR-107 for further study. Then we cloned the 3′UTR of E2F1 mRNA containing miR-107 binding site (termed pGL3-E2F1) and its mutant (termed pGL3-E2F1-mut) (supporting Figure 4A). Luciferase reporter gene assays showed that the overexpression of miR-107 suppressed the luciferase activities of pGL3-E2F1 in HepG2 cells (Figure 5A), but failed to work for pGL3-E2F1-mut in HepG2 and H7402 cells (Figure 5B). Western blot analysis showed that miR-107 was able to decrease the expression of E2F1, while the miR-107 inhibitor could block the event in the cells (Figure 5C), suggesting that miR-107 inhibits the expression of E2F1 by targeting the 3′UTR of E2F1 mRNA. In addition, the expression levels of SPHK1 were determined by Western blot analysis in HepG2 cells transfected with miR-107. We found that miR-107 was able to decrease the expression of SPHK1 (supporting Figure 4B), suggesting that miR-107 decreases the expression of SPHK1 by targeting E2F1 mRNA 3′UTR.

Given that HULC was capable of sequestering the miRNA to influence the gene expression [9], we observed that HULC potentially interacted with miR-107 through complementary base-pairs by bioinformatics analysis (http://bibiserv.techfak.uni-bielefeld.de/rnahybrid/submission.html). Accordingly, we supposed that HULC might inhibit miR-107, displaying a role of sponge. We constructed the mutant of HULC (named HULC-107-mut) to confirm the interaction between HULC and miR-107 (Figure 5D). Luciferase reporter gene assays showed that HULC could abolish the decrease of pGL3-E2F1 luciferase activities mediated by miR-107 in HepG2 cells, but HULC-107-mut failed to work (Figure 5E), suggesting that the interaction between HULC and miR-107 is associated with the event. Furthermore, we validated that the expression levels of HULC were negatively correlated with those of miR-107 in above 60 HCC tissues (*P* < 0. 001, *r* = −0.604, Pearson's correlation) (Figure 5F). In addition, we observed that the overexpression of HULC resulted in the down-regulation of miR-107 in hepatoma cells (supporting Figure 4C), suggesting that the interaction of HULC with miR-107 results in the decrease of miR-107. Meanwhile, we validated that the overexpression of miR-107 also resulted in the decrease of HULC in hepatoma cells (supporting Figure 4D). Thus, we conclude that HULC is able to up-regulate E2F1 by sequestering miR-107.

### HULC promotes tumor angiogenesis *in vitro* and *in vivo*

**Figure 6:**
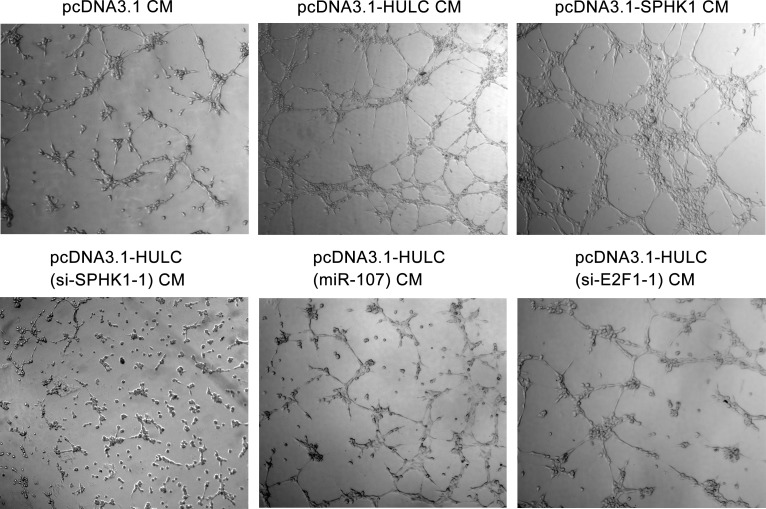
HULC accelerates tumor angiogenesis *in vitro* Representative examples of tube formation were shown as a low-power image (magnification, ×10) when HUVECs were cultured with conditioned medium.

**Figure 7:**
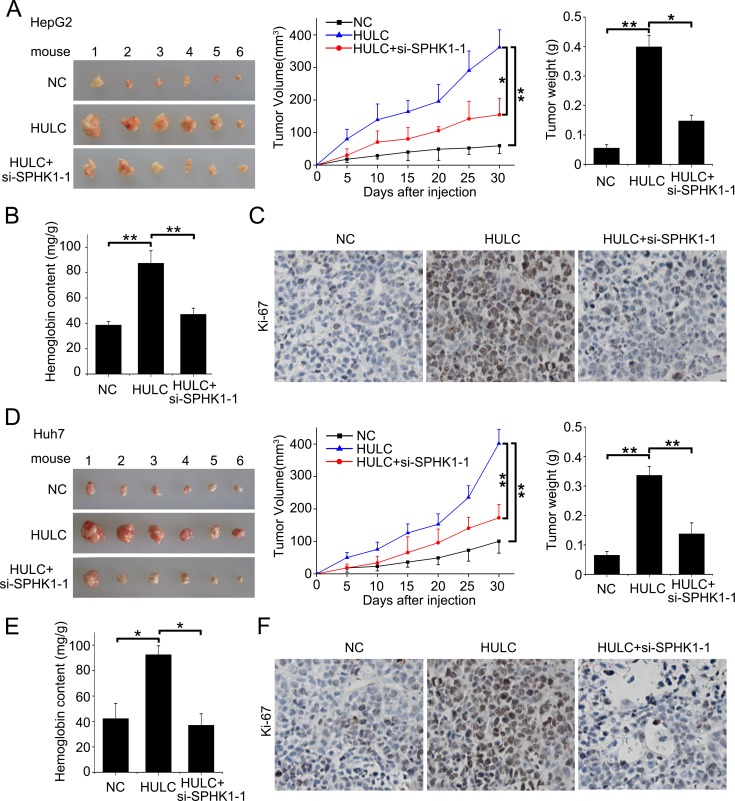
HULC promotes tumor angiogenesis *in vivo* **A.** The photographs, growth curve and weight of tumors from nude mice transplanted with HepG2 cells pretreated with pcDNA3.1 and si-control, pcDNA3.1-HULC and si-control, and pcDNA3.1-HULC and si-SPHK1-1, respectively. **B.** The levels of hemoglobin content in the tumor tissues were detected by ELISA assays. **C.** The expression of Ki-67 in the tumor tissues from mice was detected by IHC. **D.** The photographs, growth curve and weight of tumors from nude mice transplanted with Huh7 cells pretreated with pcDNA3.1 and si-control, pcDNA3.1-HULC and si-control, or pcDNA3.1-HULC and si-SPHK1-1, respectively. **E.** The levels of hemoglobin content in the tumor tissues were detected by ELISA assays. **F.** The expression of Ki-67 in the tumor tissues from mice was detected by IHC. Statistically significant differences are indicated: *P < 0.05, **P < 0.01, Student's *t* test.

Next, we evaluated the role of HULC in tumor angiogenesis *in vitro* by human umbilical vein endothelial cells (HUVECs) tube formation assays. Our data showed that the tube formation was remarkably increased when the HUVECs were cultured with 50% (v/v) conditioned medium from HULC-transfected (or SPHK1-transfected) HepG2 cells for 6 h (Figure 6 top), but the treatment with si-SPHK1-1 (or miR-107 or si-E2F1-1) obviously abrogated the HULC-enhanced tube formation in the system (Figure 6 bottom), suggesting that HULC contributes to the angiogenesis through HULC/miR-107/E2F1/SPHK1 signaling. Additionally, we identified the pro-angiogenesis function of S1P, product of SPHK1, by HUVEC tube formation assays *in vitro*. We observed that S1P could obviously promote the tube formation of HUVEC (supporting Figure 5). Then, we verified the data *in vivo* using tumor xenograft model. We found that HULC accelerated the tumor growth derived from HepG2 (or Huh7) cells in nude mice, but the treatment with si-SPHK1-1 could markedly block the tumor growth (Figure 7A and 7D). Meanwhile, the silence efficiency of HULC (or SPHK1) was confirmed by qRT-PCR and Western blot analysis in the tumor tissues from mice (supporting Figure 6A-6D). Congruously, ELISA showed that the hemoglobin content in tumor tissues derived from HULC-overexpressed group was higher than that from SPHK1-knockdown group (Figure 7B and 7E), suggesting that HULC enhances tumor angiogenesis through SPHK1. In addition, the staining of Ki-67, a marker of proliferation, further attested that the HULC-induced tumor grew faster than control group (Figure 7C and 7F), suggesting that HULC facilitated-tumor angiogenesis accelerates the growth of hepatoma *in vivo*. Taken together, we conclude that HULC promotes tumor angiogenesis *in vitro* and *in vivo.*

## DISCUSSION

LncRNAs are implicated in physiologic and pathologic processes in mammals. The dysregulation of lncRNAs is exhibited in many cancers and the levels of certain lncRNAs are associated with the development, metastasis, and prognosis of cancer [28, 29]. In this study, we are interested in the role of lncRNA HULC in the angiogenesis of liver cancer.

Given that HULC contributed to the proliferation and abnormal lipid metabolism of hepatoma cells [6, 11], we speculated that HULC might contribute to the tumor angiogenesis. We screened some important genes which were involved in the tumor angiogenesis in HULC-overexpressed HepG2 cells. Strikingly, we observed that the mRNA levels of SPHK1 could be dose-dependently elevated by HULC. We then showed that the expression levels of SPHK1 were higher in clinical hepatoma tissues relative to non-tumor tissues. In addition, we found that the levels of HULC were positively correlated with those of SPHK1 mRNA as well as its production S1P in clinical HCC tissues. It has been reported that SPHK1 is involved in the oncogenesis, the promotion of cellular survival, proliferation and transformation [30], and the stimulation of angiogenesis [19, 21]. Therefore, we supposed that HULC might promote angiogenesis through SPHK1 in liver cancer. Then, we further validated that HULC increased the level of S1P, product of SPHK1, in the medium of HepG2 cells. Interestingly, we demonstrated that HULC was capable of accelerating angiogenesis by CAM assay, in which si-SPHK1-1 remarkably abolished the event. It has been reported that lncRNA mg3 is able to inhibit tumor angiogenesis [31]. LncRNA MALAT1 can inhibit endothelial cell function and vessel growth [32]. LncRNA MVIH is capable of activating tumor-inducing angiogenesis by inhibiting the secretion of phosphoglycerate kinase 1 [33]. Therefore, our finding is consistent with the reports that lncRNAs contribute to the modulation of tumor angiogenesis.

To better understand the mechanism, we identified the core region of SPHK1 promoter. Interestingly, we found that HULC was able to activate SPHK1 promoter through transcription factor E2F1. HULC increased the expression of E2F1 in hepatoma cells and the levels of HULC were positively related to those of E2F1 in clinical HCC samples. According to the report that HULC was capable of sequestering miRNA to influence the gene expression, we conjectured that HULC might increase the expression of E2F1 in the same way. Bioinformatics analysis showed that miR-107 potentially bound 3′UTR of E2F1 mRNA. As expected, we revealed that miR-107 was able to bind the 3′UTR of E2F1 mRNA in hepatoma cells, resulting in the down-regulation of E2F1. MiR-107 was decreased in tumorigenesis, such as glioma and breast cancer [34, 35]. The transcription factor E2F1 is involved in the up-regulation of many classic oncogenes, such as c-Myc in liver cancer [36]. Thereby, we concerned whether HULC blocked miR-107. Interestingly, bioinformatics analysis showed that HULC was able to interact with miR-107 (Figure 5D), potentially displaying a sponge role. Then, we constructed the mutant of HULC, replacing the binding site of miR-107, to confirm the interaction between HULC and miR-107. Our data showed that HULC could abolish the decrease of pGL3-E2F1 luciferase activities mediated by miR-107 in HepG2 cells, but HULC-107-mut failed to work. It suggests that the interaction between HULC and miR-107 is responsible for the up-regulation of E2F1. It has been reported that lncRNA-ATB can sequester miR-200s to promote the invasion-metastasis cascade in HCC [37]. Therefore, the model of sponge which lncRNA interacts with other miRNA may be one of the important regulatory mechanisms in cells. In this study, we demonstrated the role of HULC in tumor angiogenesis in *vitro* and *in vivo*. SPHK1 may serve as a therapeutic target in liver cancer.

In summary, in this study we report that HULC is able to promote the tumor angiogenesis through miR-107/E2F1/SPHK1 signaling in liver cancer. HULC sequesters miR-107, resulting in the up-regulation of E2F1. HULC activates the transcription of SPHK1 through transcriptional factor E2F1 in hepatoma cells, leading to up-regulation of SPHK1. Thus, our finding provides new insights into the mechanism of tumor angiogenesis.

## MATERIALS AND METHODS

### Patient samples

Sixty HCC tissues utilized in this study were obtained from Tianjin First Central Hospital (Tianjin, China) after surgical resection. Clinical information about patients was obtained from patient records and summarized in supporting Table 1. All patients were diagnosed with primary HCC, and none had received previous radiotherapy or chemotherapy before surgery. Written consents approving the use of their tissues for research purposes after the operation were obtained from each patient. The study protocol was approved by the institute research ethics committee at Nankai University (Tianjin, China).

### Cell lines and cell culture

Hepatoma cell lines HepG2, Huh7 and HepG2.2.15 (a HepG2 cell line integrated full-length HBV DNA), and human embryonic kidney cell line 293T were maintained in Dulbecco's modified Eagle's medium (Gibco, Grand Island, NY, USA). H7402 cell lines was cultured in RPMI Medium 1640 (Gibco) supplemented with 10% fetal calf serum (FCS), 100 U/ml penicillin, and 100 mg/ml streptomycin in 5% CO_2_ at 37°C. HUVECs were maintained as described previously [38].

### RNA extraction, reverse-transcription and quantitative real-time polymerase chain reaction (qRT-PCR)

Total RNA was extracted from cells (or tumor tissues from mice and patient tissues) using Trizol reagent (Invitrogen). The total extraction was treated with DNase I (Sigma, USA). First-strand cDNA was synthesized as reported before [39]. To analyze miRNA-107 expression, total RNA was polyadenylated by poly (A) polymerase (Ambion, Austin, TX, USA) as described previously [39]. Reverse transcription was performed using poly (A)-tailed total RNA and reverse transcription primer with ImPro-II Reverse Transcriptase (Promega, Madison, WI, USA) according to the manufacturer's protocol. QRT-PCR was performed by a Bio-Rad sequence detection system according to the manufacturer's instructions using double-stranded DNA-specific Fast Start Universal SYBR Green Master (Roche, Indianapolis, IN, USA). Experiments were conducted in duplicate in three independent assays. Relative transcriptional folds were calculated as 2^−ΔΔCt^ [40]. GAPDH was used as an internal control for normalization. U6 was used as an internal control to normalize miRNA-107 levels. All the primers used are listed in supporting Table 3.

### S1P extraction and enzyme linked immunosorbent assay (ELISA)

The tumor tissues were homogenized and sonicated in the following homogenization buffer: 20 mM Tris-HCl, pH 7.4; 20% glycerol; 1 mM β-mercaptoethanol; 1 mM EDTA; 1 mM Naorthovanadate; 15 mM NaF; 1 mM PMSF; protease inhibitor cocktail (Sigma, USA); 0.5 mM deoxypyridoxine; 40 mM β-glycerophosphate. Apply tumor tissues homogenate samples prepared in homogenization buffer to the S1P ELISA Kit (Echelon Biosciences, Salt Lake City, UT, USA).

### Western blot analysis

Western blot was carried out using the protocol described previously [10]. The following primary antibodies were used: rabbit anti-SPHK1 (Proteintech), rabbit anti-E2F1 (Proteintech), mouse anti-β-actin (NeoMarkers, USA).

### Short interfering RNA (siRNA) and microRNA (MiRNA)

The siRNA duplexes targeting HULC, E2F1 and SPHK1 were designed and synthesized by Ribobio Co. Lit. (Guangzhou, China) as previously described [10, 41, 42]. The control cells were treated with 100nM si-Control synthesized by Ribobio Co. Lit. MiR-107 mimics and its inhibitor (anti-miR-107) and their respective negative controls were from Ribobio Co. Lit. All sequences are listed in supporting Table 4.

### Chicken chorioallantoic membrane (CAM) assays

Fertilized white leghorn chicken eggs (Gallus gallus) were incubated for 3 days at 37°C in a humidified atmosphere [43]. After 3 days, a small opening was made in the shell, which was covered afterwards with cellophane tape before the eggs were returned to the incubator. Two days later, the chorioallantoic membrane was incubated with a circular filter paper piece (5mm diameter) containing 50 μl conditional medium. Following 6 days of incubation, the eggs were opened and fixation with stationary solution (methanol: acetone=1:1) for 15 min. The pictures were taken with a stereomicroscope (Leica M165FC, Germany) [44].

### Luciferase reporter gene assays

Luciferase reporter gene assay was performed using the Dual-Luciferase Reporter Assay System (Promega) according to the manufacturer's instructions. Cells were transferred into 24-well plates at 3× 10^4^ cells per well. After 24 h, the cells were transiently co-transfected with 0.1 μg/well of pRL-TK plasmid (Promega) containing the Renilla luciferase gene used for internal normalization, and various constructs containing different lengths of the SPHK1 5′-flanking region or pGL3-Basic. The luciferase activities were measured as previously described [45]. All experiments were performed at least three times.

### Chromatin immunoprecipitation (ChIP)

ChIP assays were performed in HepG2 cells and clinical HCC tissues as reported previously [39]. The promoter region of the SPHK1 including the E2F1 sites was amplified from the immunoprecipitated DNA samples with the specific primers. All primers are listed in supporting Table 3.

### Electrophoretic mobility shift assay (EMSA)

The EMSA was carried out as described previously [45]. The nuclear extracts were obtained from HepG2 cells. Probes were generated by annealing single strand oligonucleotides containing the E2F1 consensus sequence of SPHK1 promoter and labeling the ends with [γ-^32^P] ATP using T4 polynucleotide kinase (TaKaRa Bio). The oligonucleotide sequences are listed in supporting Table 3. The primary antibodies were: rabbit anti-E2F1 (Proteintech), mouse anti-IgG antibody (Santa Cruz).

### Human umbilical vein endothelial cells (HUVEC) tubule formation assay

HUVEC tubule formation was performed as described previously [43]. Briefly, 200 μl Matrigel (BD Biosciences, Franklin Lakes, NJ, USA) was pipetted into each well of a 24-well plate and polymerized for 30 min at 37^°^C. 2× 10^4^ HUVECs in 200 μl 50% conditioned medium (CM) were added to each well and incubated at 37^°^C, 5% CO_2_ for 6 h. Images were taken in a 100× bright-field microscope. Each condition was assessed at least in triplicate in three independent experiments.

### Statistical analysis

Each experiment was repeated at least three times. Statistical significance was assessed by comparing mean values (± standard deviation, SD), using a Student's *t* test for independent groups, and was assumed for *P* < 0.05, *P* < 0.01, and *P* < 0.001. Pearson's correlation coefficient was used to determine the correlation between the levels of SPHK1 (or S1P, or E2F1, or miR-107) and HULC mRNA in HCC tissues.

## SUPPLEMENTARY MATERIAL FIGURES AND TABLES


