# Supplementary material for: Long non-coding RNA HULC promotes tumor angiogenesis in liver cancer by up-regulating sphingosine kinase 1 (SPHK1)
Source: Oncotarget. 2015 Nov 2;7(1):241–54. doi: 10.18632/oncotarget.6280 (PMC4807995; doi:10.18632/oncotarget.6280)
Supplement: Supplementary file 1 [file oncotarget-07-0241-s001.pdf]

Long non-coding RNA HULC promotes tumor angiogenesis in liver cancer by up-regulating sphingosine kinase 1 (SPHK1)

Supplementary Material

Supporting materials and methods

Plasmid construction

The 5' flanking region (nucleotides -1531 to +202) of SPHK1 was amplified by PCR from the genomic DNA of HepG2 using specific primers and cloned into the upstream of the pGL3-Basic vector (Promega) via KpnI and HindⅢ sites. The resulting plasmid was sequenced and named pGL3-1733. To construct various lengths luciferase reporter plasmids of SPHK1 promoter, the regions (-1531/-951, -970/+202, -500/+202, -300/+202, -300/+20, +1/+202) of SPHK1 were amplified from pGL3-1733, then they were inserted into the pGL3-Basic vector to generate pGL3-1172, pGL3-581, pGL3-702, pGL3-502, pGL3-320, pGL3-202, respectively. Mutant construct of pGL3-320, named as pGL3-320-mut, which had a deletion of eight nucleotides (-147 to -140) within the binding sites of E2F1, was carried out using overlap extension PCR. 3′UTR of E2F1 mRNA containing miR-107 target was cloned into pGL3-control, termed pGL3-E2F1. HULC with a deletion of miR-107 binding site was constructed to pcDNA3.1 (termed HULC-107-mut). All primers are listed in Supporting Table 3.

Cell transfection

The cells were cultured in a 6-well or 24-well plate for 24 h and then transfected with plasmids, miRNA or siRNAs, respectively. All transfections were performed using Lipofectamine 2000 reagent (Invitrogen, Carlsbad, CA, USA) according to the

manufacturer's protocol. siRNA oligonucleotides, including targeting HULC (or SPHK1 and E2F1) and a non-specific scrambled control, miR-107 (or anti-miR-107), and a miRNA control were synthesized by RiboBio (Guangzhou, China). The siRNA duplexes sequences are all listed in Supporting Table 4.

Tumor xenograft in mice and hemoglobin content analysis

Four-week-old male BALB/c athymic nude mice (Experiment Animal Center of Peking, China) (each group, n=6) were subcutaneously injected with 4×10^5 HepG2 (or Huh7) cells transfected with pcDNA3.1 and si-control (named NC), or pcDNA3.1-HULC and si-control, or pcDNA3.1-HULC and si-SPHK1-1, respectively. The growth of solid tumors after 5 days from the injected cells was monitored every five days for up to 30 days. The animals were sacrificed to remove tumors for analysis. The tumors were measured and calculated with the formula $(L \times W^2) \times 0.5$. Hemoglobin concentration was determined using the method described previously [1]. The use of mice was approved by the Animal Care and Use Committee of Nankai University.

Immunohistochemistry

Immunohistochemical staining of samples were performed as previously reported [2]. The HCC tissue and normal liver tissue microarrays (NO. 03C03) were obtained from the Xi'an Aomei Biotechnology Co., Ltd. (Xi'an, China). These microarrays were composed of 143 HCC tissues, 5 paratumor liver tissues (supporting Table 2). The primary antibody of rabbit anti-SPHK1 (Proteintech Group, USA) or anti-E2F1 (Proteintech Group, USA) was

used. The extents of cytosolic and nuclear staining were considered in the scoring. Categorization of immunostaining intensity was performed by three independent observers.

References

1. Liu F, You X, Wang Y, Liu Q, Liu Y, Zhang S, Chen L, Zhang X and Ye L. The oncoprotein HBXIP enhances angiogenesis and growth of breast cancer through modulating FGF8 and VEGF. Carcinogenesis. 2014; 35(5):1144-1153.
2. Zhang X, Dong N, Yin L, Cai N, Ma H, You J, Zhang H, Wang H, He R and Ye L. Hepatitis B virus X protein upregulates survivin expression in hepatoma tissues. J Med Virol. 2005; 77(3):374-381.

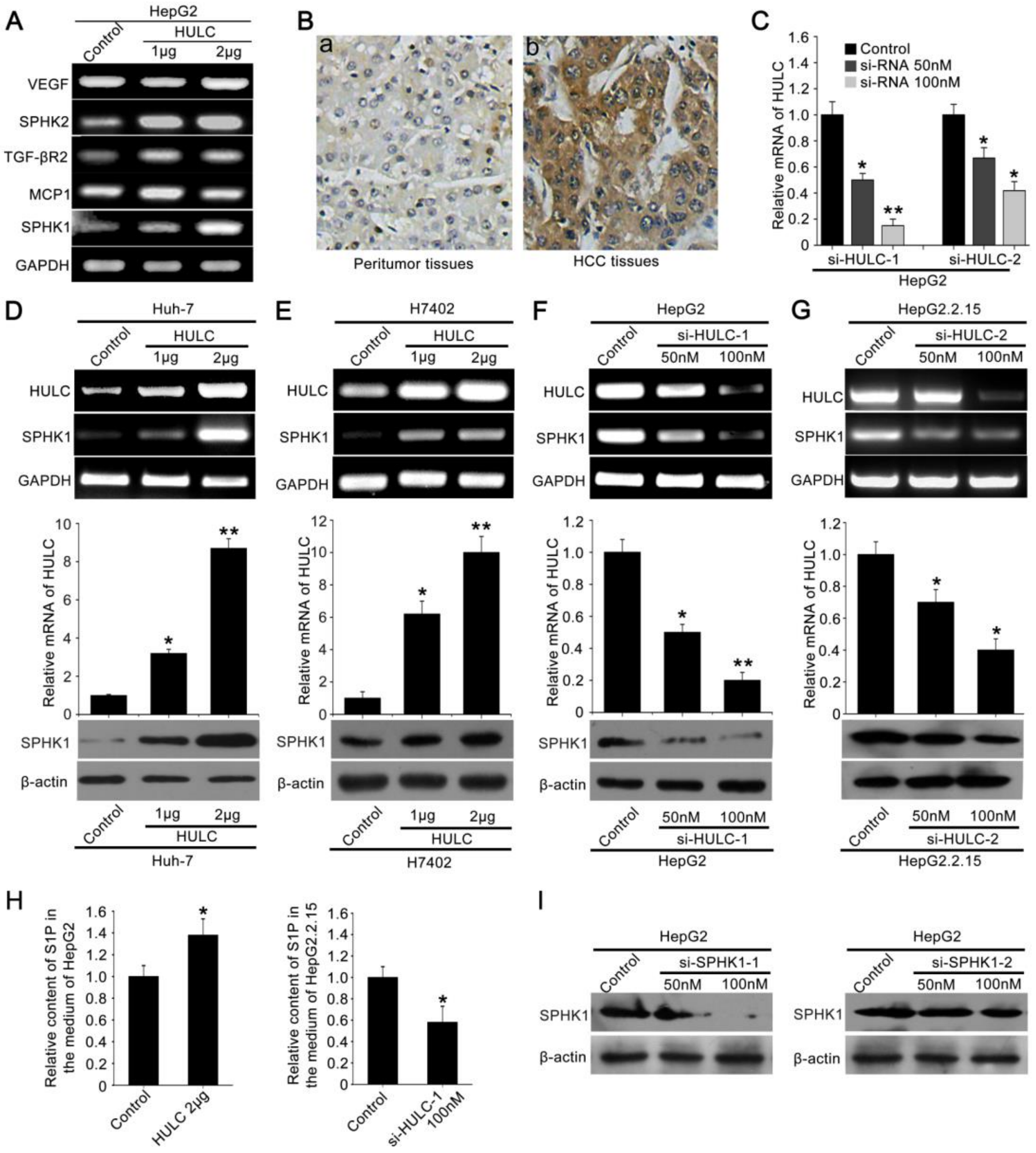

Figure S1: HULC is able to up-regulate SPHK1. (**A**) The effect of HULC on the expression of candidate angiogenic genes was examined by RT-PCR in HepG2 cells. (**B**) The expression of SPHK1 was examined by IHC staining in clinical tissues. A representative example of IHC observed in peritumoral tissues (a) and HCC tissues (b) using rabbit anti-SPHK1 Ab is presented. (**C**) The interference efficiency of si-HULC-1 and si-HULC-2 was detected by qRT-PCR. (**D, E**) The expression levels of SPHK1 were detected by RT-PCR and Western blot analysis in Huh7 (or H7402) cells transfected with pcDNA3.1-HULC. (**F, G**) The expression levels of SPHK1 were detected by RT-PCR and

Western blot analysis in HepG2 (or HepG2.2.15) cells transfected with HULC siRNA. (**H**) The relative level of S1P in the medium of HepG2 (HepG2.2.15) cells was measured by ELISA. (**I**) The interfering efficiency of si-SPHK1-1 and si-SPHK1-2 was detected by Western blot analysis in HepG2 cells. (*$P < 0.05$, **$P < 0.01$; Student's *t* test)

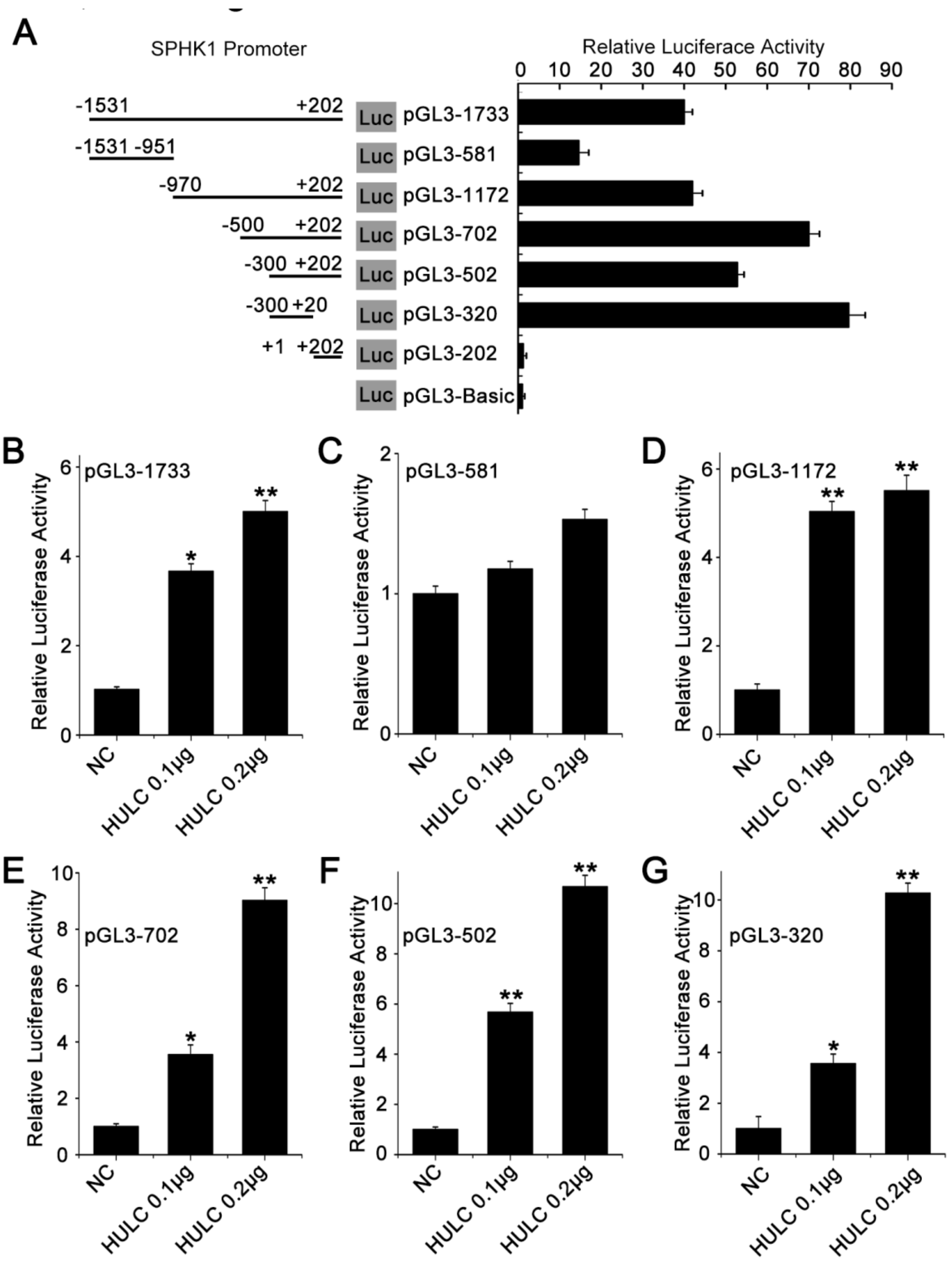

Figure S2: HULC is able to activate the promoter of SPHK1. (**A**) 293T cells were transiently transfected with pGL3-Basic (0.2 μg/well) or reporter constructs containing various lengths of the 5' -flanking region of the SPHK1 gene, as indicated (pGL3-1733, pGL3-581, pGL3-1172, pGL3-702, pGL3-502 and pGL3-320 0.2 μg/well, respectively). Results were obtained as relative luciferase activity against the activity of pGL3-Basic. (**B-G**) 293T cell lines were co-transfected with reporter constructs (0.2 μg/well) and HULC

expression plasmid (pcDNA3.1-HULC), respectively. Promoter activities of SPHK1 were measured by luciferase reporter gene assays. Data are shown as mean ± SD of three independent experiments. (*$P < 0.05$; **$P < 0.01$; Student's *t* test)

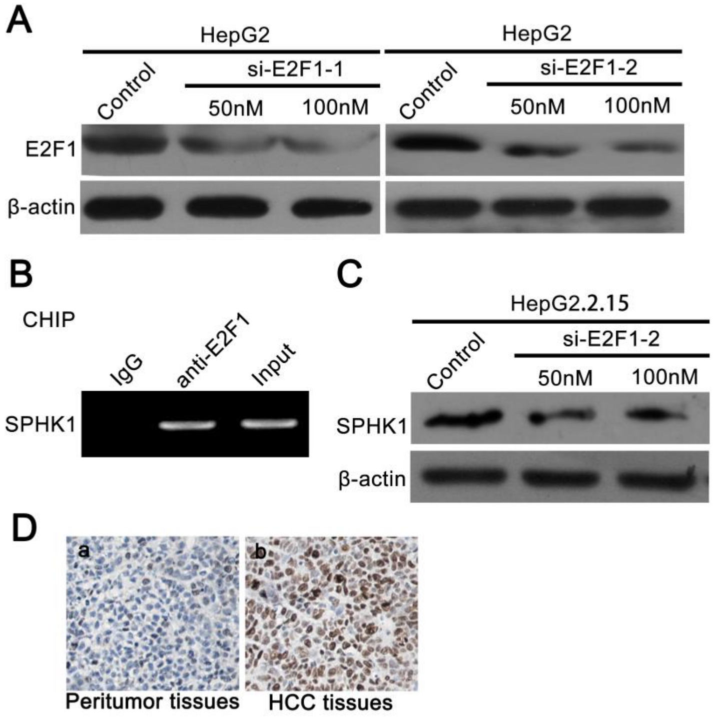

Figure S3: HULC activates SPHK1 promoter through transcription factor E2F1. (**A**) The interfering efficiency of si-E2F1-1 and si-E2F1-2 was detected by Western blot in HepG2 cells. (**B**) The interaction of E2F1 and SPHK1 promoter region was validated by ChIP assays using clinical HCC tissues. (**C**) The expression levels of SPHK1 were detected by Western blot in HepG2.2.15 cells transfected with si-E2F1-2. (**D**) The expression of E2F1 was examined by IHC staining in clinical tissues using tissue microarray. A representative example of IHC observed in peritumoral tissues (a) and HCC tissues (b) using rabbit anti-E2F1 Ab is presented.

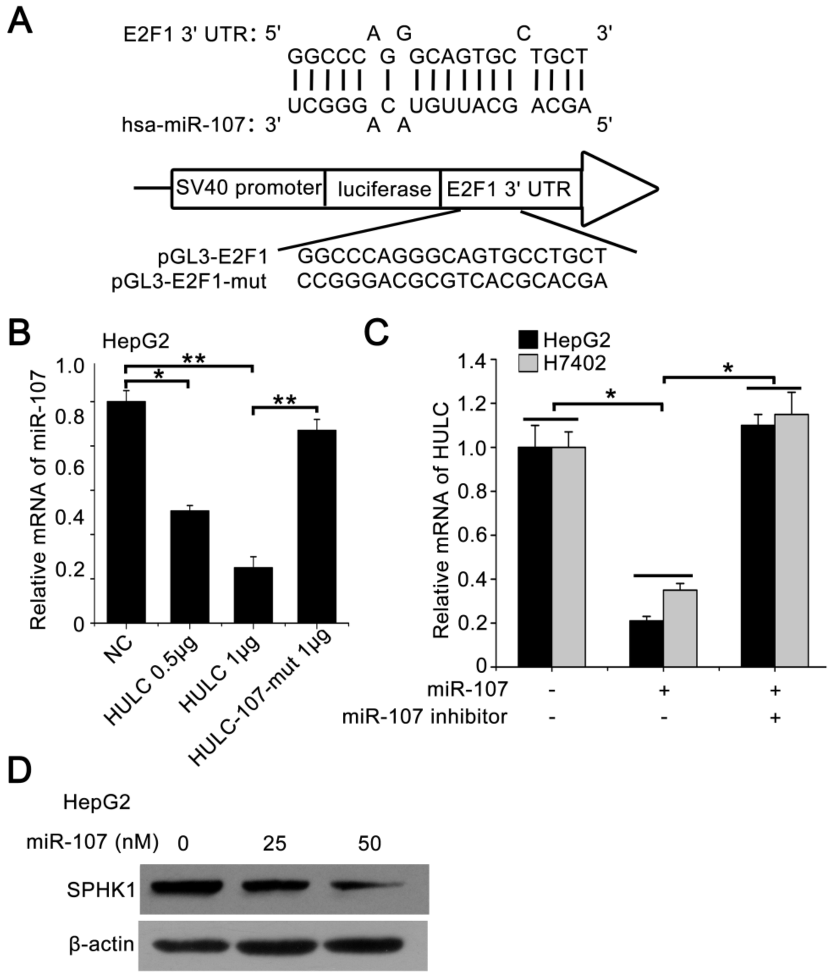

Figure S4: HULC increases E2F1 by sequestering miR-107. (A) The model demonstrated the predicted conserved miR-107 binding site at nucleotides 1872-1895 of the E2F1 3′UTR. The generated mutant site at the E2F1 3′UTR is indicated. The wild type E2F1 3′UTR (or mutant) was inserted into the downstream of luciferase reporter gene in the pGL3-Control vector. (**B**) The expression levels of SPHK1 were examined by Western blot analysis in HepG2 cells transfected with miR-107. (**C**) MiR-107 was examined by qRT-PCR in HepG2 cells transfected with pcDNA3.1, pcDNA3.1-HULC or pcDNA3.1-HULC-107-mut. (**D**) The expression levels of HULC were tested by qRT-PCR in HepG2 (or H7402) cells treated with miR-107 or miR-107 and miR-107 inhibitor. Data are shown as mean ± SD of three independent experiments. (*$P < 0.05$; **$P < 0.01$; Student's *t* test)

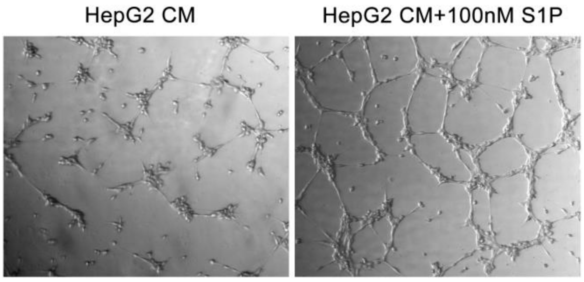

Figure S5: HULC accelerates tumor angiogenesis *in vitro.* Representative examples of tube formation were shown as a low-power image (magnification, ×10) when HUVECs were cultured with conditioned medium of HepG2 cells or conditioned medium of HepG2 cells with 100 nM S1P.

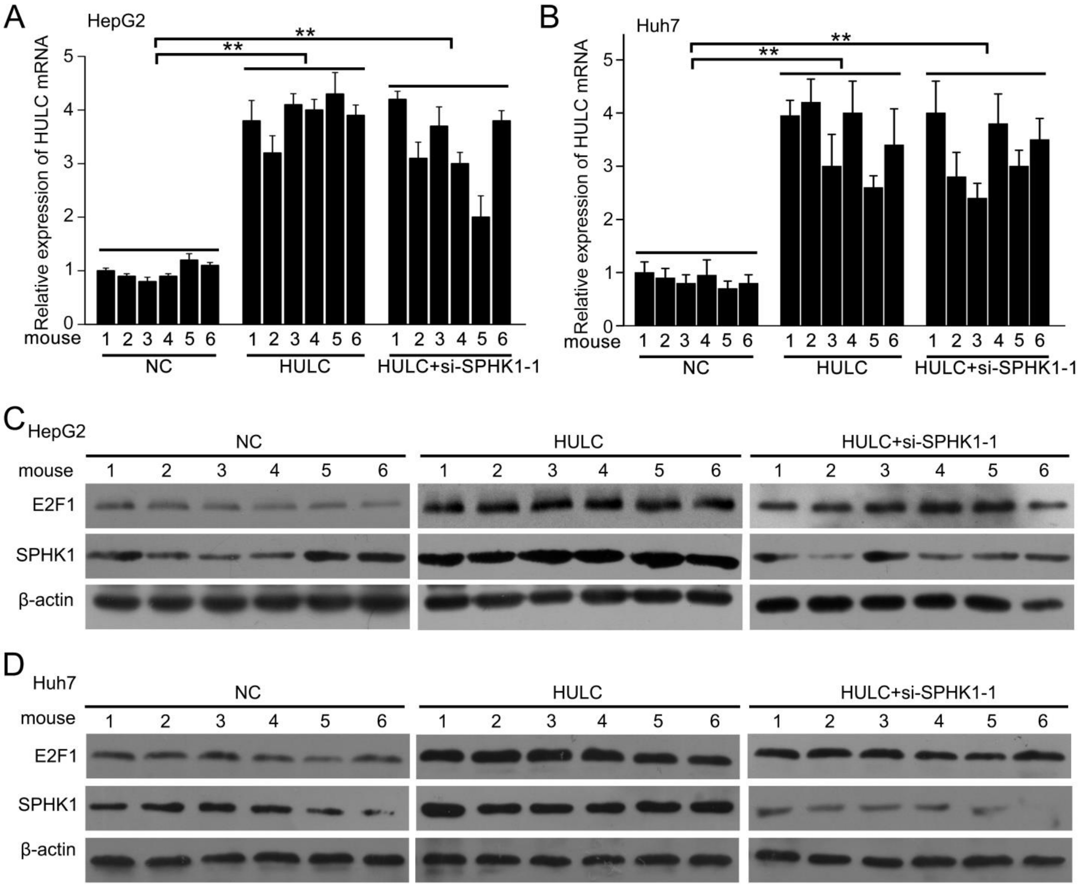

Figure S6: HULC promotes tumor angiogenesis *in vivo.* (**A, B**) The expression levels of HULC were detected by qRT-PCR in the tumor tissues transplanted with HepG2 (or Huh7) cells pretreated with pcDNA3.1 and si-control, pcDNA3.1-HULC and si-control, or pcDNA3.1-HULC and si-SPHK1. (**C, D**) The protein levels of E2F1 and SPHK1 in the tumors were detected by Western blot analysis, respectively. (**P* < 0.05; ***P* < 0.01; Student's *t* test)

Supporting Table 1: characteristics of HCC patients

Case No.	Age (yr)	Gender	Diagnosis	HBV Infection	Survival(m)
1	55	M	HCC	+	3
2	45	M	HCC	+	3
3	40	M	HCC	+	8
4	60	F	HCC	+	9
5	73	M	HCC	+	11
6	46	M	HCC	+	N/A
7	60	M	HCC	+	N/A
8	59	M	HCC	+	N/A
9	57	F	HCC	+	N/A
10	51	M	HCC	+	N/A
11	38	M	HCC	+	N/A
12	56	M	HCC	+	N/A
13	49	M	HCC	+	N/A
14	58	F	HCC	+	N/A
15	46	M	HCC	+	N/A
16	56	M	HCC	+	N/A
17	54	M	HCC	+	N/A
18	39	M	HCC	+	N/A
19	64	M	HCC	+	N/A
20	61	M	HCC	+	N/A
21	59	F	HCC	+	N/A
22	60	M	HCC	+	N/A
23	65	M	HCC	+	N/A
24	43	M	HCC	+	N/A
25	60	F	HCC	+	N/A
26	41	M	HCC	+	N/A
27	45	M	HCC	+	N/A
28	56	M	HCC	+	N/A
29	70	M	HCC	+	N/A
30	67	M	HCC	+	N/A
31	59	M	HCC	+	N/A
32	57	M	HCC	+	N/A
33	61	F	HCC	+	N/A
34	51	F	HCC	+	N/A
35	56	M	HCC	+	N/A
36	60	M	HCC	+	N/A
37	54	M	HCC	+	N/A
38	36	M	HCC	+	N/A

39	62	F	HCC	-	N/A
40	57	M	HCC	+	N/A
41	45	M	HCC	+	N/A
42	54	M	HCC	+	N/A
43	68	M	HCC	+	N/A
44	52	M	HCC	+	N/A
45	68	F	HCC	+	N/A
46	59	M	HCC	+	N/A
47	63	M	HCC	+	N/A
48	61	M	HCC	+	N/A
49	56	F	HCC	-	N/A
50	35	F	HCC	+	N/A
51	40	M	HCC	-	N/A
52	75	M	HCC	-	N/A
53	53	M	HCC	+	N/A
54	60	F	HCC	+	N/A
55	26	M	HCC	+	N/A
56	51	M	HCC	+	N/A
57	72	F	HCC	-	N/A
58	43	M	HCC	+	N/A
59	26	M	HCC	+	N/A
60	56	M	HCC	+	N/A

Abbreviations: “yr” refers to year. “m” refers to month. “N/A” refers not available

Supporting Table 2: clinical characteristics of liver cancer and normal tissue

No	Age	Sex	Organ	Pathology diagnosis	Grade	Grade of SPHK1	Grade of E2F1
**1**	55	F	Liver	Hepatocellular carcinoma	II	+	++
**2**	32	F	Liver	Hepatocellular carcinoma	I	++	++
**3**	38	M	Liver	Hepatocellular carcinoma	II	+	+
**4**	63	F	Liver	Hepatocellular carcinoma	I	++	+
**5**	35	M	Liver	Hepatocellular carcinoma	I	–	+
**6**	42	F	Liver	Hepatocellular carcinoma	I	+	++
**7**	46	M	Liver	Hepatocellular carcinoma	I	+	+
**8**	40	M	Liver	Hepatocellular carcinoma	I	+	+
**9**	48	F	Liver	Hepatocellular carcinoma	I	–	+
**10**	40	M	Liver	Hepatocellular carcinoma	I	–	–
**11**	41	F	Liver	Hepatocellular carcinoma	I	+	+
**12**	49	M	Liver	Hepatocellular carcinoma	I	–	+
**13**	42	M	Liver	Hepatocellular carcinoma	I	–	+
**14**	18	M	Liver	Hepatocellular carcinoma	I	+	–
**15**	55	M	Liver	Hepatocellular carcinoma	I	++	+
**16**	71	M	Liver	Hepatocellular carcinoma	II	+	++
**17**	43	F	Liver	Hepatocellular carcinoma	II	–	+
**18**	50	M	Liver	Hepatocellular carcinoma	I	+	+
**19**	98	F	Liver	Hepatocellular carcinoma	II	+	++
**20**	48	F	Liver	Hepatocellular carcinoma	III	+	++
**21**	58	M	Liver	Hepatocellular carcinoma	II	+	+
**22**	70	M	Liver	Hepatocellular carcinoma	II	++	++
**23**	49	M	Liver	Hepatocellular carcinoma	III	++	++
**24**	52	M	Liver	Hepatocellular carcinoma	II	++	++
**25**	56	M	Liver	Hepatocellular carcinoma	II	++	++
**26**	58	M	Liver	Hepatocellular carcinoma	II	++	++
**27**	61	M	Liver	Hepatocellular carcinoma	II	+	++
**28**	50	M	Liver	Hepatocellular carcinoma	II	+	++
**29**	39	M	Liver	Hepatocellular carcinoma	II	–	–
**30**	37	F	Liver	Hepatocellular carcinoma	II	++	++
**31**	45	M	Liver	Hepatocellular carcinoma	II	–	+
**32**	56	M	Liver	Hepatocellular carcinoma	II	–	+
**33**	64	M	Liver	Hepatocellular carcinoma	II	++	++
**34**	69	F	Liver	Hepatocellular carcinoma	II	+	++
**35**	58	M	Liver	Hepatocellular carcinoma	II	++	+
**36**	47	M	Liver	Hepatocellular carcinoma	II	–	+

**37**	49	F	Liver	Hepatocellular carcinoma	II	–	+
**38**	48	M	Liver	Hepatocellular carcinoma	II	+	–
**39**	49	M	Liver	Hepatocellular carcinoma	II	+	++
**40**	43	M	Liver	Hepatocellular carcinoma	II	+	+
**41**	42	M	Liver	Hepatocellular carcinoma	II	++	+
**42**	35	M	Liver	Hepatocellular carcinoma	II	++	++
**43**	50	M	Liver	Hepatocellular carcinoma	II	–	+
**44**	40	M	Liver	Hepatocellular carcinoma	II	+	+
**45**	33	M	Liver	Hepatocellular carcinoma	II	+	+
**46**	57	M	Liver	Hepatocellular carcinoma	III	++	+
**47**	55	M	Liver	Hepatocellular carcinoma	II	–	++
**48**	36	F	Liver	Hepatocellular carcinoma	II	–	+
**49**	63	M	Liver	Hepatocellular carcinoma	II	++	+
**50**	19	M	Liver	Hepatocellular carcinoma	II	+	++
**51**	68	M	Liver	Hepatocellular carcinoma	II	+	++
**52**	40	M	Liver	Hepatocellular carcinoma	II	–	–
**53**	52	F	Liver	Hepatocellular carcinoma	II	–	–
**54**	38	M	Liver	Hepatocellular carcinoma	II	+	++
**55**	48	M	Liver	Hepatocellular carcinoma	II	–	+
**56**	53	M	Liver	Hepatocellular carcinoma	II	+	–
**57**	41	M	Liver	Hepatocellular carcinoma	II	–	+
**58**	35	F	Liver	Hepatocellular carcinoma	II	–	–
**59**	27	M	Liver	Hepatocellular carcinoma	III	–	++
**60**	65	M	Liver	Hepatocellular carcinoma	II	+	++
**61**	39	F	Liver	Hepatocellular carcinoma	II	+	+
**62**	41	M	Liver	Hepatocellular carcinoma	II	+	+
**63**	46	M	Liver	Hepatocellular carcinoma	II	+	+
**64**	60	M	Liver	Hepatocellular carcinoma	II	++	++
**65**	41	M	Liver	Hepatocellular carcinoma	II	+	++
**66**	45	M	Liver	Hepatocellular carcinoma	II	+	+
**67**	48	F	Liver	Hepatocellular carcinoma	II	–	+
**68**	47	M	Liver	Hepatocellular carcinoma	II	++	++
**69**	47	M	Liver	Hepatocellular carcinoma	II	+	++
**70**	75	M	Liver	Hepatocellular carcinoma	II	–	+
**71**	25	M	Liver	Hepatocellular carcinoma	II	–	–
**72**	51	M	Liver	Hepatocellular carcinoma	III	++	++
**73**	55	M	Liver	Hepatocellular carcinoma	II	+	+
**74**	65	M	Liver	Hepatocellular carcinoma	II	++	++

**75**	45	M	Liver	Hepatocellular carcinoma	II	–	–
**76**	46	M	Liver	Hepatocellular carcinoma	II	+	+
**77**	46	M	Liver	Hepatocellular carcinoma	II	–	+
**78**	43	M	Liver	Hepatocellular carcinoma	II	++	++
**79**	62	F	Liver	Hepatocellular carcinoma	III	–	++
**80**	35	M	Liver	Hepatocellular carcinoma	II	++	++
**81**	47	F	Liver	Hepatocellular carcinoma	II	++	++
**82**	47	M	Liver	Hepatocellular carcinoma	II	–	++
**83**	67	M	Liver	Hepatocellular carcinoma	II	++	++
**84**	63	M	Liver	Hepatocellular carcinoma	II	+	+
**85**	45	M	Liver	Hepatocellular carcinoma	II	++	+
**86**	52	F	Liver	Hepatocellular carcinoma	II	–	++
**87**	48	F	Liver	Hepatocellular carcinoma	II	++	–
**88**	37	M	Liver	Hepatocellular carcinoma	III	++	++
**89**	69	F	Liver	Hepatocellular carcinoma	I	–	+
**90**	60	M	Liver	Hepatocellular carcinoma	II	–	++
**91**	62	F	Liver	Hepatocellular carcinoma	II	+	–
**92**	70	M	Liver	Hepatocellular carcinoma	II	++	++
**93**	40	M	Liver	Hepatocellular carcinoma	II	+	++
**94**	45	M	Liver	Hepatocellular carcinoma	II	++	++
**95**	40	M	Liver	Hepatocellular carcinoma	II	+	+
**96**	74	M	Liver	Hepatocellular carcinoma	II	+	+
**97**	45	M	Liver	Hepatocellular carcinoma	II	+	++
**98**	48	F	Liver	Hepatocellular carcinoma	II	–	–
**99**	40	M	Liver	Hepatocellular carcinoma	II	++	+
**100**	47	M	Liver	Hepatocellular carcinoma	III	++	++
**101**	32	M	Liver	Hepatocellular carcinoma	II	+	++
**102**	58	M	Liver	Hepatocellular carcinoma	II	+	+
**103**	47	M	Liver	Hepatocellular carcinoma	II	–	–
**104**	26	M	Liver	Hepatocellular carcinoma	II	–	+
**105**	65	F	Liver	Hepatocellular carcinoma	II	+	+
**106**	52	M	Liver	Hepatocellular carcinoma	III	+	++
**107**	54	M	Liver	Hepatocellular carcinoma	II	–	+
**108**	48	M	Liver	Hepatocellular carcinoma	II	+	+
**109**	63	M	Liver	Hepatocellular carcinoma	II	++	–
**110**	63	M	Liver	Hepatocellular carcinoma	III	–	+
**111**	67	M	Liver	Hepatocellular carcinoma	III	+	–
**112**	43	M	Liver	Hepatocellular carcinoma	III	+	++

**113**	46	M	Liver	Hepatocellular carcinoma	III	_	+
**114**	35	M	Liver	Hepatocellular carcinoma	III	++	+
**115**	38	M	Liver	Hepatocellular carcinoma	III	+	++
**116**	58	M	Liver	Hepatocellular carcinoma	III	++	++
**117**	41	M	Liver	Hepatocellular carcinoma	III	++	++
**118**	56	M	Liver	Hepatocellular carcinoma	III	++	++
**119**	72	M	Liver	Hepatocellular carcinoma	III	++	++
**120**	65	M	Liver	Hepatocellular carcinoma	III	+	++
**121**	56	M	Liver	Hepatocellular carcinoma	III	++	++
**122**	38	M	Liver	Hepatocellular carcinoma	III	++	++
**123**	43	F	Liver	Hepatocellular carcinoma	III	_	++
**124**	51	M	Liver	Hepatocellular carcinoma	I	_	_
**125**	51	M	Liver	Hepatocellular carcinoma	III	++	+
**126**	52	M	Liver	Hepatocellular carcinoma	III	_	+
**127**	68	M	Liver	Hepatocellular carcinoma	III	++	_
**128**	52	M	Liver	Hepatocellular carcinoma	III	+	++
**129**	50	M	Liver	Hepatocellular carcinoma	III	+	_
**130**	49	M	Liver	Hepatocellular carcinoma	III	_	_
**131**	56	F	Liver	Hepatocellular carcinoma	III	_	+
**132**	68	M	Liver	Hepatocellular carcinoma	III	_	+
**133**	55	M	Liver	Hepatocellular carcinoma	III	+	+
**134**	54	M	Liver	Hepatocellular carcinoma	III	_	_
**135**	62	M	Liver	Hepatocellular carcinoma	III	+	+
**136**	53	M	Liver	Hepatocellular carcinoma	III	_	_
**137**	66	M	Liver	Hepatocellular carcinoma	III	++	+
**138**	56	M	Liver	Hepatocellular carcinoma	III	++	+
**139**	32	F	Liver	Hepatocellular carcinoma	III	+	++
**140**	47	M	Liver	Hepatocellular carcinoma	III	+	++
**141**	39	M	Liver	Hepatocellular carcinoma	III	_	++
**142**	56	F	Liver	Hepatocellular carcinoma	III	++	+
**143**	68	M	Liver	Hepatocellular carcinoma	III	_	+
**144**	38	F	Liver	Normal	—	+	+
**145**	60	M	Liver	Normal	—	_	_
**146**	46	M	Liver	Normal	—	_	_
**147**	63	M	Liver	Normal	—	_	+
**148**	63	M	Liver	Normal	—	_	_

Supporting Table 3: primers used in the paper

Gene	Primer	Sequence (5′-3′)
Primers for SPHK1 promoter		
pGL3-1733	forward	CGGGGTACCCTCGGAGGTGCAGGACCCAT
pGL3-1172	forward	CGGGGTACCTGGCAACTTCTTCCTCCGTC
pGL3-702	forward	CGGGGTACCCTCGAATTTCGGGTGGGCTA
pGL3-502	forward	CGGGGTACCCGCGCGCCCCAGACACTGCG
pGL3-202	forward	CGGGGTACCAGTGCCCTCCCCGCTCCGCG
	reverse	CCCAAGCTTCTACCCAGTCGGTCCGGTTT
pGL3-581	forward	CGGGGTACCCTCGGAGGTGCAGGACCCAT
	reverse	CCCAAGCTTGACGGAGGAAGAAGTTGCCA
pGL3-320	forward	CGGGGTACCCGCGCGCCCCAGACACTGCG
	reverse	CCCAAGCTTCGCGGAGCGGGGAGGGCACT
Primers for qRT-PCR		
HULC	forward	ATGGGGGTGGAACTCATGATGG
	reverse	AAGAATGGACATCATTTTATTTCA
SPHK1	forward	CTGTCACCCATGAACCTGCT
	reverse	TACAGGGAGGTAGGCCAGTC
E2F1	forward	AAACAAGGCCCGATCGATGT
	reverse	GGTGGGGAAAGGCTGATGAA
GAPDH	forward	GGGAGCCAAAAGGGTCATCA
	reverse	TGATGGCATGGACTGTGGTC
miRNA-107	forward	AGCAGCATTGTACAGGGCTATCA
	reverse	GCGAGCACAGAATTAATACGAC
U6	forward	AGAGCCTGTGGTGTCCG
	reverse	CATCTTCAAAGCACTTCCCT
VEGF	forward	ACTGCCATCCAATCGAGACC
	reverse	CAGGGCATTAGACAGCAGCG
SPHK2	forward	TCGTTCTGTGTCTGACCTGC
	reverse	CATGAGCACAAAGTCCCCCT
TGF-βR2	forward	TAGGACTGCCCATCCACTGA
	reverse	GAGGCTGATGCCTGTCACTT
MCP1	forward	CTCGCTCAGCCAGATGCAAT
	reverse	TTCTTTGGGACACTTGCTGC
Primers for CHIP		
	forward	CAGACGCCTAGGACGAGC
	reverse	CCGGGGGTGGAACCTGA
Nucleotide sequence for EMSA (-152/-135)		
	forward	CCCGGGCGGGAACCAGCT
	reverse	AGCTGGTTCCCGCCCGGG
E2F1 3'UTR	forward	GCTCTAGACGGGGAATGAAGGTGAACATAC
	reverse	GGGGGCCGGCCTATGGGGCAGAAGAACAGCTCA
E2F1 3'UTR-mut	forward	CTGGGGGTCCCACAACCGGGTCCCGTCAC GGACGTCCCAGAATCTGGTG

	reverse	CACCAGATTCTGGGACGTCCGTGACGGGA CCCGGTTGTGGGACCCCCAG
HULC-107-mut	forward	AAATTTGTACTTTCTGGGACTTAATACAAC AAATCAAAGAAAAAAAT
	reverse	ATTTTTTTCTTTGATTTGTTGTATTAAGTCC CAGAAAGTACAAATTT

Supporting Table 4: list of siRNA and miRNA mimics

Gene		Sequence (5′-3′)
**siRNA Duplexes**		
si-HULC-1	sense	CCUCCAGAACUGUGAUCCAdTdT
si-HULC-2	sense	AGCUCUUGUCUCUUCUUCCCdTdT
si-E2F1-1	sense	UGGACCACCUGAUGAAUAUdTdT
si-E2F1-2	sense	UUUGUUCUCCGAAGAGUCCACdTdT
si-SPHK1-1	sense	GGGCAAGGCCUUGCAGCUCUdTdT
si-SPHK1-2	sense	AUACUUCUCACUCUCUAGGUCdTdT
Negative control	sense	UUCUUCGAAGGUGUGACGUdTdT
has-miR-107	sense	AGCAGCAUUGUACAGGGCUAUCA
miR-107 inhibitor	sense	UGAUAGCCCUGUACAAUGCUGCU
